# Biological age in the meta omics era: Integrating physical activity, sleep, and diet to reshape paths of healthy ageing

**DOI:** 10.1002/imo2.70127

**Published:** 2026-07-30

**Authors:** Yanwei You, Yicong Cui, Yuquan Chen, Jiarong He, Dongmin Wang, Danijela Gasevic, Xifeng Wu, Steve Horvath, Mingming Zhang, Xindong Ma

**Affiliations:** ^1^ Saw Swee Hock School of Public Health National University of Singapore Singapore Singapore; ^2^ Division of Sports Science & Physical Education Tsinghua University Beijing China; ^3^ School of Sports Science and Engineering East China University of Science and Technology Shanghai China; ^4^ School of Public Health and Preventive Medicine, Faculty of Medicine, Nursing & Health Sciences, Monash University Melbourne Australia; ^5^ Department of Neurosurgery The Second Xiangya Hospital, Central South University Changsha China; ^6^ Hunan Cerebrospinal Vascular Disease Diagnosis and Treatment Center, The Second Xiangya Hospital, Central South University Changsha China; ^7^ Department of Physical Education Peking University Beijing China; ^8^ Centre for Global Health, Usher Institute The University of Edinburgh Edinburgh UK; ^9^ School of Public Health, Li Ka Shing Faculty of Medicine The University of Hong Kong Hong Kong China; ^10^ Center of Clinical Big Data and Analytics of The Second Affiliated Hospital, and School of Public Health Zhejiang University School of Medicine Hangzhou China; ^11^ Human Genetics, David Geffen School of Medicine University of California Los Angeles California USA; ^12^ IDG/McGovern Institute for Brain Research Tsinghua University Beijing China

## Abstract

Biological age provides a translational endpoint linking modifiable lifestyle behaviors—physical activity, sleep, and diet—to multi‐omics changes and downstream clinical outcomes. Integrating multiple ageing clocks with inflammatory, metabolic, and oxidative pathways supports reproducible, intervention‐ready strategies to slow ageing and extend healthspan.

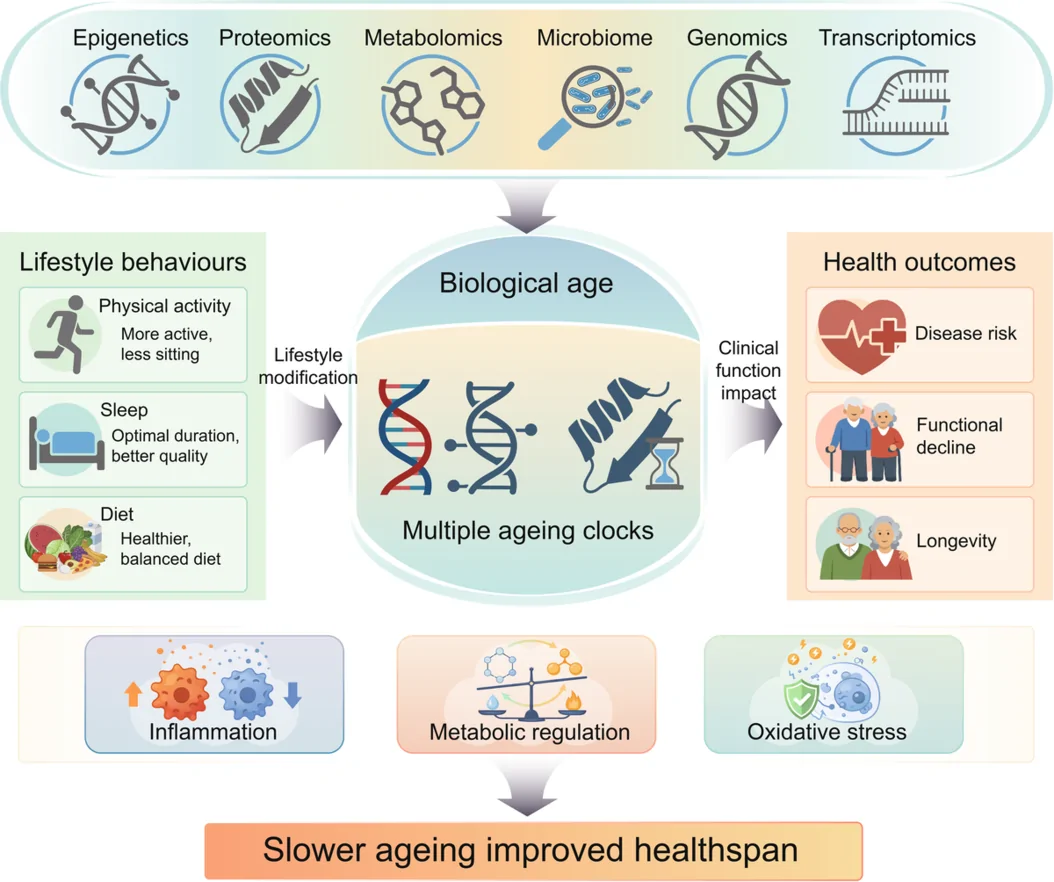

1

The field of healthy ageing is undergoing a visible shift from a disease‐centered paradigm to a function‐centered and resilience‐centered model. Chronological age remains indispensable for describing population structure, yet it is increasingly insufficient for explaining why people of the same age diverge so sharply in functional decline, multimorbidity, and survival [1]. This heterogeneity reflects the long‐term imprint of modifiable behaviors and environments layered onto genetic susceptibility, and it also challenges the conventional practice of relying on age‐stratification or a single age‐adjustment term to solve biological complexity. A biologically informed time‐scale is therefore needed to move healthy ageing research beyond descriptive associations toward actionable models that connect lifestyle patterns to molecular change and clinically meaningful endpoints [2].

The term “Biological age” was proposed to capture an individual's ageing status using measurable biological features rather than chronological time, offering an intuitive framework [3]. Over the past two decades, methodological advances have enabled scalable quantification through composite biomarker approaches and omics‐based clocks. To facilitate interpretation, these measures can be classified into distinct categories. *Clinical biomarker clocks*: Among widely used measures, Phenotypic Age (PhenoAge) integrates chronological age with a weighted panel of routine biochemical and hematological indicators reflecting inflammation, metabolic health, renal/hepatic function, and hematopoietic health—to better stratify morbidity and mortality risk in large cohorts than chronological age alone [4]. *DNA‐methylation clocks*: Epigenetic clocks derived from DNA methylation signatures represent a landmark development, providing higher‐resolution estimators of ageing. For interpretability, they can be organized into “chronological DNAm age” measures (e.g., HorvathAge, HannumAge) that primarily track calendar time, and “health/risk & pace” measures (e.g., PhenoAge, GrimAge, DunedinPACE) calibrated to mortality/morbidity risk or longitudinal pace of ageing. Second‐generation clocks such as DNAm GrimAge further strengthen clinical relevance by incorporating methylation surrogates for plasma proteins and smoking history [5]. *Proteomic and metabolomic clocks*: Beyond DNA methylation, large‐scale plasma proteome and metabolome analyses show systematic age‐related shifts, supporting the construction of signatures relevant to disease risk and physiological integrity [6, 7, 8]. *Gut Microbiome clocks*: More recently, gut microbiome‐derived estimators have been proposed to capture ageing‐related changes in host–microbe ecology, which may relate to systemic inflammation and metabolic phenotypes [9].

Despite their potential, no single clock fully captures multi‐system ageing, and their broad application faces several challenges. Measurement harmonization and standardization remain a major challenge for reproducibility and for making results comparable across cohorts. Precision ageing studies require robust computation of biological age measures with appropriate quality control and batch correction to address calibration issues. Furthermore, addressing ancestry‐ and setting‐specific bias in biological age inference remains critical as populations diversify.

To address these challenges and advance precision healthy ageing, it is essential to integrate meta‐omics, biological age, and lifestyle factors. The overarching aim of this review is to articulate the rationale for this integration. Figure 1 summarizes the conceptual model: positioning biological age as a primary endpoint, or as a mediator linking lifestyle exposures to functional outcomes, offers several practical advantages for healthy ageing research. First, many biological age measures provide stronger prediction of downstream outcomes than chronological age alone, including disability, frailty‐relevant phenotypes, multimorbidity, and all‐cause mortality, which is central for translating observational evidence into clinically meaningful risk stratification. Second, biological age measures are more sensitive to cumulative environmental and behavioral influences, supporting analyses that quantify how physical activity, sleep patterns, and diet quality relate to multi‐system ageing burden rather than isolated disease endpoints. Third, biological age is well‐suited to repeated‐measurement designs: within‐person change over time can be modeled as a dynamic outcome, enabling stronger longitudinal inference about temporal ordering and responsiveness to intervention.

**Figure 1:**
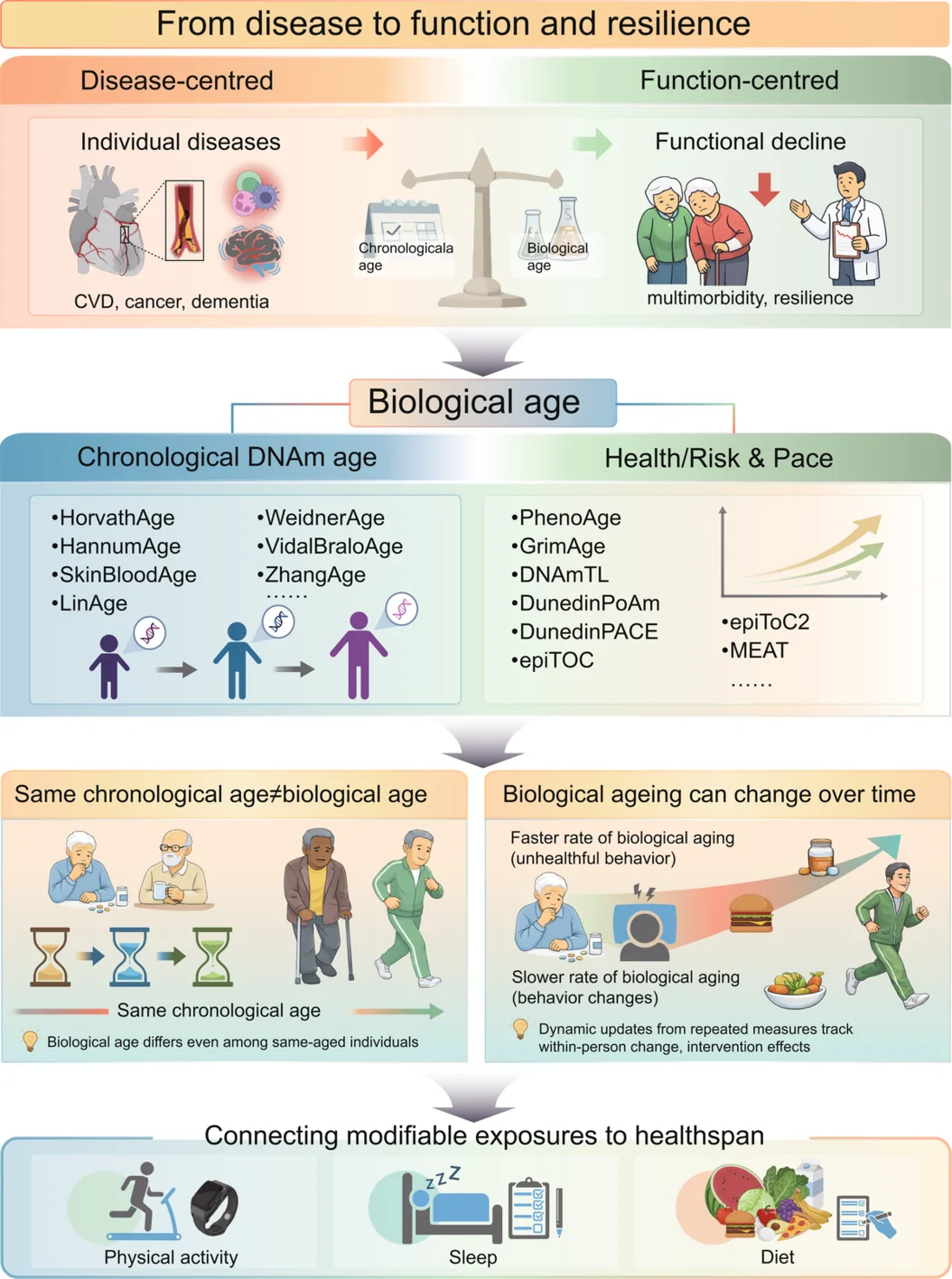
Schematic overview: biological age as a translational bridge from disease to function and resilience. Biological age links disease‐centered outcomes with function‐centered endpoints by integrating chronological DNAm clocks and health‐, risk‐, and pace‐related ageing measures. The framework highlights inter‐individual differences at the same chronological age, dynamic changes over time, and the role of modifiable exposures, including physical activity, sleep, and diet, in shaping healthspan. CVD, cardiovascular disease; DNAm, DNA methylation; DNAm age, DNA methylation age; DNAmTL, DNA methylation‐based telomere length estimator; DunedinPoAm, Dunedin Pace of Aging methylation algorithm; DunedinPACE, Dunedin Pace of Aging Calculated from the Epigenome; epiTOC, Epigenetic Timer of Cancer; epiTOC2, Epigenetic Timer of Cancer‐2; GrimAge, DNA methylation GrimAge; HorvathAge, Horvath DNA methylation age; HannumAge, Hannum DNA methylation age; LinAge, Lin DNA methylation age estimator; MEAT, Muscle Epigenetic Age Test; PhenoAge, Phenotypic Age or DNA methylation PhenoAge; SkinBloodAge, skin and blood DNA methylation age; Vidal‐Bralo DNA methylation age estimator; WeidnerAge, Weidner DNA methylation age estimator; VidalBraloAge, ZhangAge, Zhang DNA methylation age estimator.

### Mapping lifestyle determinants of biological age in the meta‐omics era

Building on the rationale for using biological age as a core endpoint, we performed a knowledge map analysis to characterize how the literature has evolved over the past two decades and to visualize the growing integration of biological age research with meta‐omics profiling and modifiable lifestyle behaviors. We searched the Web of Science Core Collection database on 10 February 2026, covering publications from 1 January 2005 to 31 December 2025; the full search strategy, inclusion criteria, and analytical procedures are provided in the supplementary file. This analysis summarizes publication growth, disciplinary distribution, and geographic and institutional contributions, and it delineates the shifting thematic structure of studies linking biological ageing with lifestyle exposures, with particular emphasis on physical activity, sleep, and diet.

Figure 2A shows a clear acceleration in annual output after 2017, followed by a second expansion phase around 2023. This temporal pattern is consistent with two shifts in the ageing science literature: first, the rapid uptake of DNA methylation clocks and their application in population cohorts, and second, the more recent consolidation of multi‐omics integration, scalable wearable technologies, and advanced causal inference designs in lifestyle epidemiology. Notably, the surge after 2023 suggests that biological age has moved from a niche methodological construct to a widely adopted outcome framework, increasingly used to quantify inter‐individual heterogeneity in ageing trajectories and to evaluate the health relevance of modifiable exposures.

**Figure 2:**
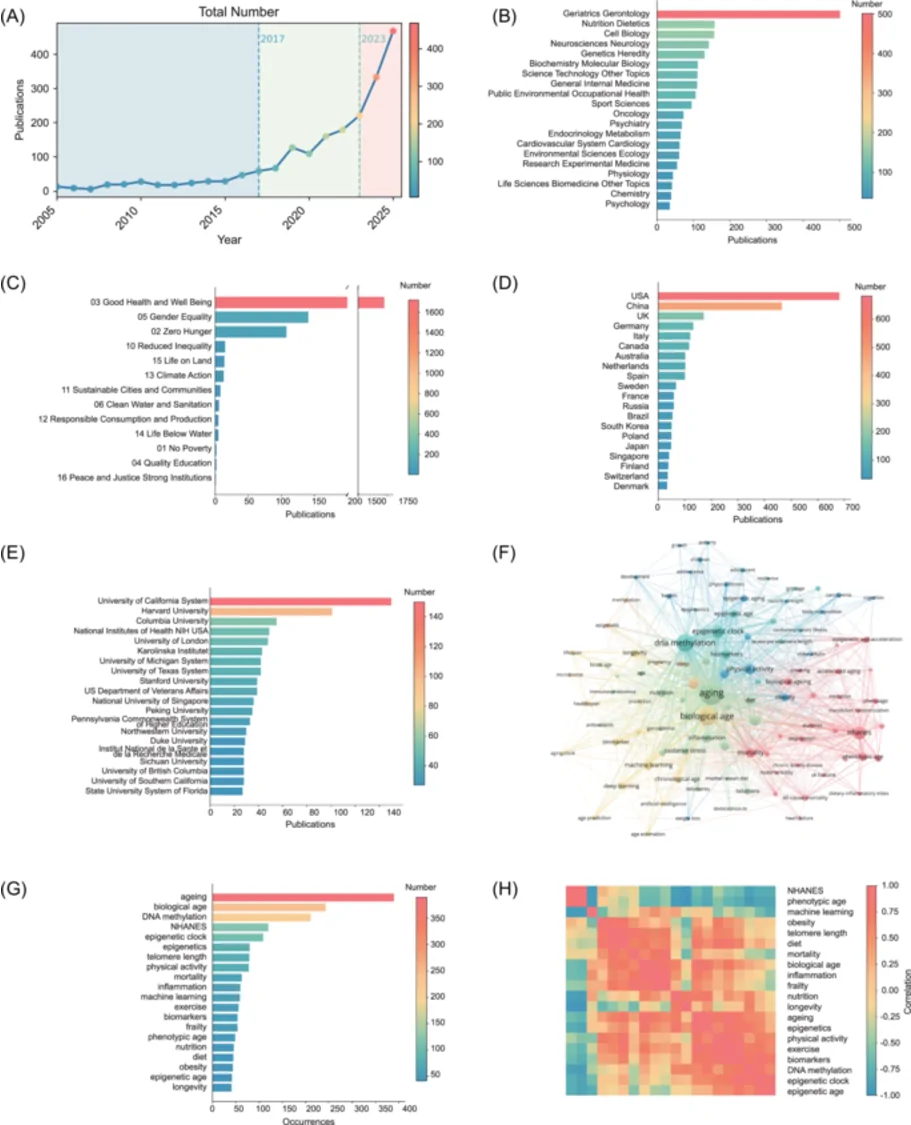
Knowledge map of the research landscape linking biological age with lifestyle behaviors in the meta‐omics era. (A) Annual publication growth (2005–2025) with inflection points highlighted; (B) dominant Web of Science subject categories; (C) mapped SDG themes represented by the literature; (D) top contributing countries by output; (E) leading institutions ranked by publication volume; (F) keyword co‐occurrence network revealing thematic clusters. In panel (F), nodes represent keywords, node size indicates occurrence frequency, links indicate co‐occurrence relationships, link thickness reflects link strength, and colors denote thematic clusters. Keywords are displayed in lower case following VOSviewer preprocessing and export to maintain consistent term normalization; (G) most frequent keywords indicating core research topics; (H) keyword correlation heatmap summarizing co‐occurrence strength across themes.

The disciplinary distribution (Figure 2B) further supports this shift toward integrative ageing science. The most productive categories are anchored in geriatrics and gerontology, followed by nutrition and dietetics, cell biology, neuroscience, genetics, and molecular biology‐related areas. Importantly, the presence of sport sciences, public health‐relevant domains, and general internal medicine indicates that biological age is not confined to molecular ageing research; rather, it is increasingly being operationalised as a measurable endpoint linking behavioral exposures, physiological dysregulation, and clinically meaningful outcomes. This breadth reflects the broader logic of the “meta‐omics era,” in which biological age metrics increasingly serve as shared endpoints that facilitate comparison across cohorts, platforms, and exposure definitions.

From a public health and sustainability perspective, the Sustainable Development Goals (SDGs) mapping (Figure 2C) demonstrates that this research domain aligns most strongly with SDG 3 (Good Health and Well‐Being), with substantial representation in SDG 5 (Gender Equality) and SDG 2 (Zero Hunger). This pattern reflects how biological age is frequently studied in the context of chronic disease prevention, health disparities, nutrition‐related risk, and sex‐specific ageing profiles. The relatively lower contribution to other SDGs does not imply irrelevance, but rather indicates that current research is still primarily anchored in health endpoints and risk stratification, with fewer studies framed explicitly around environmental sustainability, policy implementation, or cross‐sectoral interventions.

Global contributions show a concentrated but international research network. Country rankings (Figure 2D) indicate that the United States and China contribute the largest volume of publications, followed by the United Kingdom and several European and Oceanian countries. This distribution is consistent with the availability of large‐scale cohorts, biobanks, and omics platforms, as well as the analytic infrastructure required for high‐dimensional ageing metrics. At the institutional level, Figure 2E shows that output is led by major research systems and universities, particularly the University of California System, Harvard University, Columbia University, the National Institutes of Health, and the University of London. The presence of institutions such as the National University of Singapore and Peking University among the leading contributors further suggests that Asian research centers are increasingly visible in this field. Singapore appears as a visible contributor in the country ranking, which is notable given its smaller population base, and likely reflects strategic investment in population health cohorts, digital health infrastructure, and translational ageing research agendas in Asian settings. Collectively, these patterns highlight both the dominance of a few high‐output regions and the growing diversification of study populations, which is essential for external validation and for reducing ancestry‐ and setting‐specific bias in biological age inference. At the same time, the geographic concentration of the literature suggests that many current biological age estimators may be developed and validated within relatively restricted ancestral and sociocultural contexts.

The thematic structure in Figure 2F–H clarifies how the field organizes knowledge and what concepts connect lifestyle exposures with ageing metrics. The keyword co‐occurrence network (Figure 2F) reveals a highly connected core centered on ageing, biological age, epigenetic age, DNA methylation, and physical activity, with peripheral clusters spanning mortality and chronic disease outcomes, inflammation and biomarkers, telomere length, diet and nutrition, and algorithmic approaches such as machine learning. This topology indicates that the literature is simultaneously advancing along two lines: refining biological age measurement (clock construction, validation, and interpretation) and expanding application domains (lifestyle, disease risk, and population health translation).

Consistent with this, the top keyword frequencies (Figure 2G) show that “ageing” and “biological age” remain dominant anchors, while “DNA methylation,” “epigenetic clock/age,” “physical activity,” and “telomere length” represent major measurement and exposure pillars. The presence of “mortality,” “frailty,” “inflammation,” and “biomarkers” further suggests that studies are increasingly expected to demonstrate clinical relevance beyond statistical association, often by linking biological age acceleration to functional decline and hard endpoints. Notably, the presence of cohort identifiers among high‐frequency terms suggests that several large public datasets have become widely used reference resources, supporting reproducibility and cross‐study comparability.

The correlation heatmap (Figure 2H) provides an interpretable summary of topic co‐appearance patterns. Strong correlations among epigenetic age, DNA methylation, and epigenetic clocks are expected and reflect shared methodological foundations. The clustering of physical activity, diet/nutrition, obesity, inflammation, and mortality suggests that lifestyle exposures are most often examined within cardiometabolic and systemic inflammation frameworks, while biological ageing endpoints are used to quantify cumulative physiological burden. Meanwhile, the co‐appearance of machine learning with multiple ageing‐related terms suggests a growing emphasis on model development, feature selection across omics layers, and predictive performance optimization. Together, these patterns highlight a field that is moving beyond single‐marker studies toward integrated models that combine behavioral exposures, multi‐omics signatures, and clinically interpretable endpoints.

### Lifestyle determinants of biological age: Physical activity, sleep, and diet

Building on the macro‐level patterns identified in our knowledge map, the next question is what lifestyle factors are most consistently linked to biological ageing and how these associations manifest across different biological age measures. Our knowledge map analysis showed that physical activity, sleep, and diet appear consistently as major themes, matching the main clusters identified in the knowledge map.

Across diverse cohorts and measurement frameworks, individuals who move more and sit less tend to exhibit younger biological age profiles, particularly when ageing is quantified using DNA methylation‐based clocks. Epidemiologic evidence generally supports an inverse association between habitual physical activity and biological age acceleration [10, 11]. Studies using objective monitoring have shown that higher accelerometer‐derived activity and lower sedentary time are associated with more favorable epigenetic ageing profiles, including slower pace‐of‐ageing measures and reduced age acceleration across DNA methylation clocks [12]. Mechanistically, these patterns are consistent with the multi‐system effects of physical activity on cardiometabolic regulation, immune function, and inflammatory tone.

Sleep duration, sleep continuity, and circadian alignment appear to be linked to biological age acceleration, with evidence suggesting that both insufficient sleep and sleep disturbance are associated with older epigenetic age. In population studies, shorter sleep and insomnia symptoms have been related to faster epigenetic ageing, supporting sleep as a relevant behavioral dimension for biological ageing phenotypes [13, 14]. Sleep loss and circadian disruption can promote chronic low‐grade inflammation, impair glucose metabolism, alter neuroendocrine regulation, and increase oxidative stress—processes that align with the core physiological domains captured by established mortality‐ and morbidity‐predictive epigenetic clocks [4, 5]. At present, the evidence is more consistent with the importance of optimal duration and quality than with a simple longer‐is‐better interpretation, and the relationship may be non‐linear.

Diet quality adds another modifiable layer to biological ageing, and its effects become most informative when interpreted alongside physical activity and sleep within a multi‐omics framework. Dietary exposures likely influence biological ageing trajectories through sustained effects on systemic inflammation, oxidative stress, mitochondrial function, and metabolic homeostasis. While the diet–clock literature is heterogeneous in measurement and confounding control, higher‐quality dietary patterns and healthier macronutrient profiles tend to associate with younger biological age, whereas adverse dietary behaviors may relate to accelerated ageing signatures [15]. Beyond observational evidence, a key methodological advance is the increasing use of multimodal lifestyle interventions to test whether coordinated improvements in diet (often combined with physical activity, stress management, or sleep optimization) can shift epigenetic age metrics over time; early trial data suggest that biological age measures may respond within months under structured programs [16].

Moreover, physical activity, sleep, and diet do not operate in isolation. In real‐world settings, these behaviors may also interact through synergistic, antagonistic, or substitution patterns, and their effects may be partly mediated by shared intermediate layers such as gut microbiome dynamics, metabolomic remodeling, and chronic low‐grade inflammation. This joint action is one reason why meta‐omics approaches are increasingly used to connect lifestyle exposures to intermediate molecular signatures and downstream biological age shifts [17]. For example, epigenetic clocks such as DNAm PhenoAge and GrimAge extend beyond chronological prediction by incorporating or correlating with multi‐system risk profiles that better reflect cumulative physiological dysregulation. At the same time, emerging clocks derived from proteomics, metabolomics, and microbiome features offer complementary windows into ageing biology and may capture exposure‐responsive signals that are not fully represented by DNA methylation alone. Integrating these clocks within harmonized analytical and hypothesis‐driven frameworks may help identify which lifestyle components are most strongly associated with biological ageing, through which molecular pathways, and in which populations.

### Perspective for precision healthy ageing research

A practical roadmap for integrating lifestyle behaviors and biological age in precision healthy ageing research begins by formalizing a coherent conceptual pathway linking modifiable lifestyle behaviors to molecular change and downstream clinical relevance. A useful organizing framework is that lifestyle behaviors may shape multi‐omics profiles, which may in turn relate to shifts in biological age and, ultimately, to differences in functional performance, morbidity burden, and survival. Positioning biological age centrally allows clearer hypotheses about exposure responsiveness, biological plausibility, and clinical translation, while enabling harmonized comparisons across heterogeneous cohorts and ageing measures.

Design choices should prioritize longitudinal evidence capable of capturing within‐person change in both exposures and biological age. Repeated measurement in cohorts provides leverage to separate stable between‐person differences from dynamic trajectories, and to evaluate whether changes in physical activity, sleep, or diet precede shifts in biological age and related omics signatures. Large‐scale wearable studies illustrate how device‐based phenotyping can produce high‐resolution behavioral measures in population settings, supporting finer‐grained modeling of dose, timing, and behavioral patterning. For example, in the UK Biobank accelerometer sub‐study [18], wrist‐worn accelerometry enabled scalable quantification of physical activity behaviors in tens of thousands of participants, highlighting feasibility for future repeated assessments and integration into ageing research pipelines. Beyond cohorts, randomized trials and pragmatic interventions remain essential, particularly when the goal is to establish behavioral effects on biological age change under controlled or real‐world conditions.

Measurement harmonization and standardization remain a major obstacle to reproducibility and to meaningful comparisons across cohorts. Precision healthy ageing studies require consistent construction of behavioral phenotypes, transparent pre‐processing of omics data, and robust computation of biological age measures with appropriate quality control and batch correction. Methodological guidance from the multi‐omics literature emphasizes that integrative designs must be aligned with biological questions and supported by rigorous analytic validation, rather than treated as purely data‐driven exercises [17, 19]. In parallel, biological age models and risk‐prediction tools should adhere to transparent reporting, external validation, and clarity on intended use [20].

For biological age measures to become clinically actionable, future work will need to move beyond high‐dimensional black‐box prediction toward more explainable biomarker panels and risk‐stratification frameworks that can be communicated, validated, and implemented in practice. Target trial emulation provides a structured approach to defining the causal question, eligibility criteria, treatment strategies, follow‐up, and outcomes in observational datasets, improving interpretability and reducing design‐related biases when randomized trials are infeasible. Mendelian randomization offers complementary evidence by using genetic variants as instruments to test causal relevance of exposures or intermediate pathways, while requiring careful assessment of assumptions and pleiotropy. At present, however, causal evidence for lifestyle exposures such as sleep and physical activity remains evolving and may still be influenced by phenotype definition, sample ancestry, and residual socioeconomic structure. These approaches can be combined with mediation and multi‐stage modeling to evaluate how much of a lifestyle effect on functional endpoints is captured through biological age change versus other mechanisms. Future work should also examine whether associations differ across population subgroups, such as individuals with different baseline biological age levels, socioeconomic backgrounds, comorbidity profiles, and ancestral backgrounds.

## AUTHOR CONTRIBUTIONS

**Yanwei You:** Conceptualization; methodology; formal analysis; writing—original draft; writing—review and editing; visualization; supervision. **Yicong Cui:** Methodology; formal analysis; data curation; writing—original draft; writing—review and editing; visualization. **Yuquan Chen:** Methodology; formal analysis; data curation; writing—original draft; writing—review and editing. **Jiarong He:** Investigation; writing—review and editing. **Dongmin Wang:** Supervision; writing—review and editing. **Danijela Gasevic:** Supervision; writing—review and editing. **Xifeng Wu:** Supervision; writing—review and editing. **Steve Horvath:** Supervision; writing—review and editing. **Mingming Zhang:** Conceptualization; supervision; project administration; funding acquisition; writing—review and editing. **Xindong Ma:** Conceptualization; supervision; project administration; funding acquisition; writing—review and editing.

## CONFLICT OF INTEREST STATEMENT

The Regents of the University of California are the sole owners of patents and patent applications directed at epigenetic biomarkers for which Steve Horvath is a named inventor. S. H. is a founder and paid consultant of the non‐profit Epigenetic Clock Development Foundation that licenses these patents. S. H. is a Principal Investigator at the Altos Labs, Cambridge Institute of Science, a biomedical company.

## ETHICS STATEMENT

No animals or humans were involved in this study.

## Supporting information


**Supplementary File 1:** Methods for knowledge map analysis.

## Data Availability

The datasets used in this study and the analysis code have been deposited in Zenodo and are publicly available at https://zenodo.org/records/20838343. Supplementary materials, including methods, graphical abstract, slides, videos, Chinese translated version, and updated materials, may be found in the online DOI or iMetaOmics (http://www.imeta.science/imetaomics/).
