# Supplementary material for: Biological age in the meta omics era: Integrating physical activity, sleep, and diet to reshape paths of healthy ageing
Source: IMetaOmics. 2026 Jul 30;3(3):e70127. doi: 10.1002/imo2.70127 (PMC13674388; doi:10.1002/imo2.70127)
Supplement: Supplementary file 1 [file IMO2-3-e70127-s001.docx]

Supporting Information to

**Biological age in the meta omics era: integrating physical activity, sleep, and diet to reshape paths of healthy ageing**

**Running title**: **Physical Activity, Sleep, Diet, and Biological Ageing**

Yanwei You ^1,2#*^, Yicong Cui ^3#^, Yuquan Chen ^4#^, Jiarong He ^5,6^, Dongmin Wang ^7^, Danijela Gasevic ^4,8,9^, Xifeng Wu ^10^, Steve Horvath ^11^, Mingming Zhang ^5,6*^, Xindong Ma ^2,12*^

^1^ Saw Swee Hock School of Public Health, National University of Singapore, Singapore, Singapore;

^2^ Division of Sports Science & Physical Education, Tsinghua University, Beijing, China;

^3^ School of Sports Science and Engineering, East China University of Science and Technology, Shanghai, China;

^4^ School of Public Health and Preventive Medicine, Faculty of Medicine, Nursing & Health Sciences, Monash University, Melbourne, Australia;

^5^ Department of Neurosurgery, The Second Xiangya Hospital, Central South University, Changsha, China;

^6^ Hunan Cerebrospinal Vascular Disease Diagnosis and Treatment Center, The Second Xiangya Hospital, Central South University, Changsha, China;

^7^ Department of Physical Education, Peking University, Beijing, China;

^8^ Centre for Global Health, Usher Institute, The University of Edinburgh, Edinburgh, United Kingdom;

^9^ School of Public Health, Li Ka Shing Faculty of Medicine, The University of Hong Kong, Hong Kong, China;

^10^ Center of Clinical Big Data and Analytics of The Second Affiliated Hospital, and School of Public Health, Zhejiang University School of Medicine, Hangzhou, China;

^11^ Human Genetics, David Geffen School of Medicine, University of California, Los Angeles, United States;

^12^ IDG/McGovern Institute for Brain Research, Tsinghua University, Beijing, China.

^#^These authors contributed equally: Yanwei You, Yicong Cui, Yuquan Chen.

^*^Correspondence: Yanwei You (yyw22@tsinghua.org.cn), Mingming Zhang (zhangmm@csu.edu.cn), and Xindong Ma (maxd@mail.tsinghua.edu.cn).

**Methods for Knowledge Map Analysis**

**Data acquisition and search strategy**

We searched the literature on 10 February 2026, covering publications from 1 January 2005 to 31 December 2025.

The search was conducted in the Web of Science Core Collection using the Topic field (TS), which retrieves terms from titles, abstracts, and keywords. The query combined biological ageing measures, including phenotypic age (PhenoAge) and DNA methylation (DNAm)-based ageing indicators, with movement–sleep–diet-related exposures:

TS = (“biological age” OR “biological aging” OR “biological ageing” OR “phenotypic age” OR PhenoAge OR “epigenetic age” OR “epigenetic clock*” OR “epigenetic age acceleration” OR “DNA methylation age” OR “DNAm age” OR “methylation age” OR “age acceleration” OR GrimAge OR “DunedinPACE” OR “DunedinPoAm” OR “Horvath clock” OR “Hannum clock”)
AND TS = (“physical activity” OR exercise OR training OR “sedentary behavio?r” OR “physical inactivity” OR “cardiorespiratory fitness” OR “aerobic fitness” OR acceleromet* OR MVPA OR sleep OR “sleep duration” OR “sleep quality” OR insomnia OR “sleep deprivation” OR “circadian rhythm” OR “circadian disruption” OR chronotype OR “sleep disorder*” OR “obstructive sleep apnea” OR diet* OR nutrition* OR “diet quality” OR “dietary pattern*” OR “Mediterranean diet” OR DASH OR “ultra-processed food*” OR fasting OR “intermittent fasting” OR “time-restricted feeding”).

Records were limited to peer-reviewed articles and reviews in English. A total of 1,983 publications are included for analysis, the details of all included publications are uploaded online (https://zenodo.org/records/20838343).

**Data processing and bibliometric indicators**

Bibliographic metadata (including publication year, author keywords, journal subject categories, author affiliations, and countries/regions) were exported with complete records for downstream analysis.

We quantified annual publication output and used the inflection points highlighted in Figure 2A to summarise temporal phases (2005–2016, 2017–2022, and 2023–2025). Publication distributions were described across Web of Science subject categories (Figure 2B). To characterise potential sustainability relevance, we mapped publications to the United Nations Sustainable Development Goals (SDGs) using keyword-based classification implemented in R and summarised counts by SDG category (Figure 2C). Geographic productivity was examined at the country level and visualised using ranked bar charts (Figure 2D). Institutional productivity was summarised by standardised affiliation names and presented as ranked outputs (Figure 2E).

To describe the conceptual structure of the field, we analysed keyword frequency and co-occurrence. High-frequency keywords were summarised by occurrences (Figure 2G). We additionally computed a keyword–keyword association matrix and visualised it as a correlation heatmap in R to show how major concepts co-vary across the literature (Figure 2H; warmer colours indicate stronger positive correlations, cooler colours indicate weaker or negative correlations).

**Knowledge-map construction and visualisation (VOSviewer)**

Network visualisation was conducted using VOSviewer. Keyword co-occurrence networks were built based on author keywords, and the resulting network was clustered using VOSviewer’s built-in mapping and clustering approach. During keyword preprocessing and network visualisation, terms were retained in a unified lower-case format to maintain consistent term normalisation across the dataset. In bibliometric mapping, different capitalisation forms of the same term, such as “dna” and “DNA”, may be interpreted as separate terms when the underlying keyword strings are not fully harmonised, potentially altering occurrence counts, clustering structure, and link-strength relationships. Therefore, all keywords were displayed in lower case in the exported VOSviewer figures to preserve the original network structure and avoid post hoc relabelling that might introduce inconsistencies. In the network map (Figure 2F), each node represents a keyword; node size reflects the number of occurrences; links indicate co-occurrence relationships; and link strength reflects how frequently two keywords appear together across the dataset. Colours denote clusters of closely related terms, allowing identification of major thematic groupings (e.g., epigenetic clocks/DNAm ageing, lifestyle exposures, and ageing-related outcomes). This lower-case presentation follows the VOSviewer preprocessing and export workflow and does not affect the interpretation of the thematic clusters.

**Software**

All descriptive statistics, data cleaning, and figure generation were performed using R version 4.5.2. Data processing was conducted mainly with the tidyverse framework, including dplyr, tidyr, stringr, and readr. Trend plots, bar charts, and heatmaps were generated using ggplot2, with graphical arrangement performed using patchwork. Keyword co-occurrence networks were constructed and visualised using VOSviewer version 1.6.20.
